# Impact of troublesome expansive weed *Rumex alpinus* on species diversity of mountain pastures in Tatra National Park, Poland

**DOI:** 10.2478/s11756-018-0148-9

**Published:** 2018-12-11

**Authors:** Anna Delimat, Piotr Kiełtyk

**Affiliations:** 1 W. Szafer Institute of Botany, Polish Academy of Sciences, Lubicz 46, 31-512 Kraków, Poland; 2 Faculty of Biology and Environmental Sciences, Cardinal Stefan Wyszyński University in Warsaw, Wóycickiego 1/3, 01-938 Warszawa, Poland

**Keywords:** Native expansive species, Species richness, Vegetation, Tatra Mountains, Western Carpathians, Poland

## Abstract

Alpine dock (*Rumex alpinus*) is a native plant species in many European mountain ranges. It comes from moist brushwood and open tall herb communities, though it has invaded mountain pastures. This troublesome weed forms permanent monodominant stands characterised by low nature conservation and agricultural values. However, to the best of our knowledge, there are no studies assessing the impact of *R. alpinus* on invaded communities. Therefore, in this study we examined the potential effect of an *R. alpinus* on species richness, diversity and the composition of invaded vegetation in Tatra National Park, Poland. Invaded plots dominated by *R. alpinus* had, compared to uninvaded control plots, significantly reduced species richness, as well as low Shannon and Simpson diversity and Pielou evenness indices’ values; the species richness decline in the pairs of invaded-uninvaded plots was as much as 37.7%. Analysing changes in diversity from an increase in *R. alpinus* coverage revealed that Shannon, Simpson and Pielou’s diversity indices’ values did not considerably change up to 30% of *R. alpinus* coverage, but a further increase in *R. alpinus* coverage led to a steep decline in diversity. There was exception in a number of species per plot, which decreased steadily along with the increase of *R. alpinus* coverage. Multivariate analysis indicated a significant shift in the composition of invaded plots in relation to uninvaded plots, resulting from the shared increase of more shadow-tolerant and higher soil base-, nutrient- and moist-demanding species.

**Electronic supplementary material:**

The online version of this article (10.2478/s11756-018-0148-9) contains supplementary material, which is available to authorized users.

## Introduction

Mountain pastures developed from long-lasting, traditional pasturage farming in European high mountain regions are considered high conservation value zoo-anthropogenic habitats. Regular grazing, along with subsequent soil fertilization from manure, have made these pastures species-rich plant communities. However, in the middle of the last century, in many central European mountains including the Tatra Mountains, pasturage practices have ceased due to various socioeconomic reasons and nature conservation restrictions, such as the establishment of protected areas (Stachurska-Swakoń 2008). Consequently abandoned or inappropriately managed mountain pastures, particularly over-fertilized locations, were invaded by alpine dock (*Rumex alpinus* L., *Polygonaceae*), a native in Poland troublesome species capable of forming permanent, monodominant stands characterised by a relatively low nature conservation and agricultural values (Spatz 1980; Rehder 1982; Bohner 2005).

Plant invasions are often reported as exerting a negative impact on the diversity and species composition of invaded plant communities (Levine et al. 2003; Hejda et al. 2009; Hejda et al. 2017). Expansive plants can displace or considerably suppress native plants through superior competitiveness, altering ecosystem processes or disturbance regimes (Levine et al. 2003; Bottollier-Curtet et al. 2013; Gaertner et al. 2014; Gruntman et al. 2014). In particular, invading species capable of forming dense populations can have a profound effect on native species diversity (Hejda et al. 2009). Despite the fact that *R. alpinus* is regarded as a troublesome weed and requires population control (Hujerová et al. 2013; Šilc and Gregori 2016), to our best knowledge there are no studies assessing the plant’s impact on species richness and diversity in invaded vegetation. Present knowledge on the species composition of *Rumex alpinus*-dominated vegetation comes from phytosociological studies documenting species composition of the *Rumicetum alpini* plant association (Šmarda 1963; Kliment and Jarolímek 1995; Stachurska-Swakoń 2008, 2009). However, these studies do not assess diversity decrease in invaded vegetation, nor the changes in species composition with the increase of *R. alpinus* coverage. The aim of this study is to examine and quantify the potential impact of *R. alpinus* on species diversity of invaded vegetation plots in abandoned mountain pastures in Tatra National Park, Western Carpathians. To accomplish this, we used two different approaches. In the first, a space-for-time substitution approach (Hejda and Pyšek 2006), we compared vegetation characteristics of plots invaded by *R. alpinus* to uninvaded plots (representing vegetation before invasion); both plots were spatially close and environmentally similar. In the second approach, diversity measures of plots invaded by *R. alpinus* were modelled by means of linear and quadratic regression as a function of *R. alpinus* coverage.

In this paper, we asked, in particular, the following questions:

1. What is the impact of *R. alpinus-*dominated stands on species richness, diversity, evenness and composition of invaded vegetation?
2. How will increasing *R. alpinus* cover influence the diversity of plant communities?
3. Which native species are most affected by *R. alpinus* dominance?

## Materials and methods

### Study species and area

*Rumex alpinus* is distributed throughout European high mountains, including the Apennines and mountains of the Balkan Peninsula and Caucasus (Meusel et al. 1965; Tutin et al. 1993). The plant has also been introduced to some European countries, including Great Britain, Scandinavian countries and areas of the Czech Republic (Št’astná et al. 2010), where it is often considered as alien invasive species (Hejda et al. 2009; Hejda and de Bello 2013; Šurinová et al. 2018). It is suggested that the natural habitats in central European mountains from which *R. alpinus* colonised secondary zoo-anthropogenic habitats are moist brushwood communities along mountain streams in the upper montane zone and open tall herb communities of the class Mulgedio-Aconitetea (Stachurska-Swakoń 2009). *R. alpinus* is a rhizomatous perennial with a horizontal rhizome growing at a depth of 5–10 cm. Because a new segment of the rhizome develops each year, the plant’s growth and age can be determined from the number of segments (Klimešová et al. 2013). It has been reported that a rhizome can be as much as 120 cm long and can even persist for 35 years (Št’astná et al. 2010; Št’astná et al. 2012). Each year, three to five big leaves grow from a rhizome segment, with petiole ranging from 70 to 80 cm and lamina up to 50 cm long and 20 cm wide, creating a dense canopy of robust, leafy shoots with a density of 3–8 m^−2^ and height of 30–200 cm. They can also produce 1500–5000 fruits (Št’astná et al. 2010). *R. alpinus* is a nitrophilic plant species associated with moist, nutrient- and base-rich soils (Rehder 1982; Bohner 2005). The plant is a strong competitor capable of forming species-poor stands, which are known to persist for several decades (Ellenberg 2009). Vegetation dominated by *R. alpinus* was described in phytosociology as the *Rumicetum alpini* Beger 1922 plant association, but a number of syntaxonomical ranks were later distinguished (Kliment and Jarolímek 1995; Stachurska-Swakoń 2009). Št’astná et al. (2010) provides a comprehensive review of the biology and ecology of *R. alpinus* in central Europe.

**Fig. 1:**
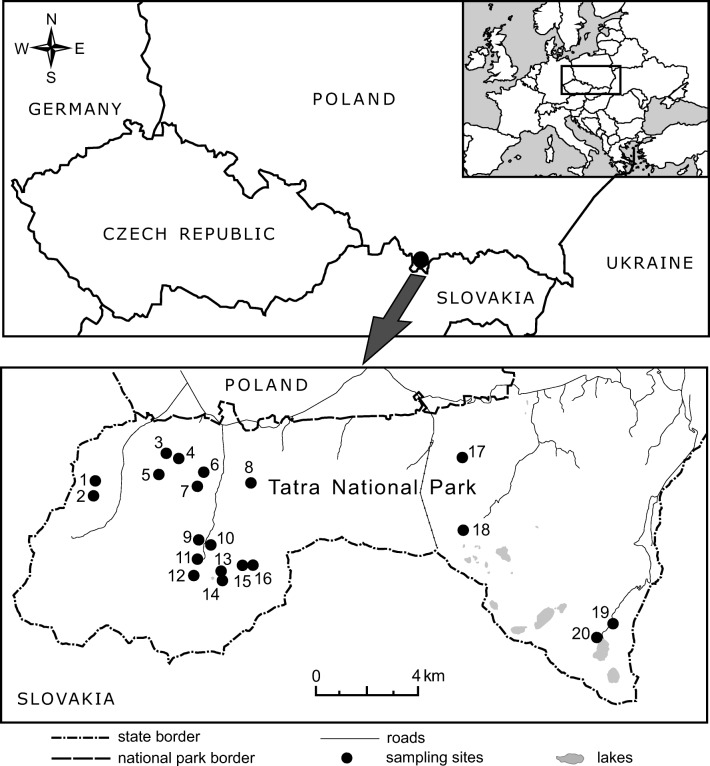
Location of the study area in Tatra National Park. Dots represent pastures where plots with *Rumex alpinus* were sampled. Numbers correspond to localities listed in Table 1

This study was conducted in the Tatra Mountains (Fig. 1) in the protected area of Tatra National Park in southern Poland. Five vertical vegetation belts are present in the Polish Tatra Mountains: the lower montane belt reaches up to 1250 m a.s.l.; the upper montane belt from 1250 to 1550 m a.s.l.; the subalpine belt from 1550 to 1800 m a.s.l.; the alpine belt from 1800 to 2300 m a.s.l.; and the sub-nival belt from 2300 m a.s.l. up (Mirek and Piękoś-Mirkowa 1992). The mean annual temperature decreases from approximately +6 °C at the foothills of the Polish Tatras (about 900–1000 m a.s.l.) to −2 °C at 2200 m a.s.l., and − 4 °C at the highest peaks. The mean annual sum of precipitation measured in the foothills at the weather station in the town of Zakopane (at an elevation of 844 m a.s.l.) is 1138 mm, and, at the weather station on the Kasprowy Wierch peak (at an elevation of 1991 m a.s.l.), 1876 mm (Hess 1996).

The beginning of pasturage management in the Tatra Mountains dates back to the thirteenth or fourteenth century. In the nineteenth and first half of the twentieth century, pasturage practices in the Tatra Mountains were intensive, with large flocks of sheep and cows causing serious devastation to mountain meadows, glades and surrounding forests (Śmiałowska 1962; Kołowca 1962). In 1947, with the establishment of a separate administrative unit, the Tatra Park, the first actions were undertaken to reduce the number of livestock in the Polish Tatras. After the establishment of the Tatra National Park in 1954, further pasturage limitations took place in the Tatra Mountains (Śmiałowska 1962). In subsequent years, pasturage was gradually ceased on alpine meadows and pastures, as well as in most glades within limits of Tatra National Park; from 1981 on, only a limited “cultural pasturing” was carried out in selected glades at lower elevations (Mielczarek 1984). A concise history outline on pasturage in Tatra National Park (with selected references) is provided by Stachurska-Swakoń (2008).

### Vegetation sampling

Data was collected in 2015 from 20 glades and pastures located in the vertical zone of the lower and the upper montane vegetation belts, where formerly pasturage took place (Fig. 1, Table 1). At these sites, we examined 100 vegetation plots with *R. alpinus* of 4 m^2^ and 100 control plots of the same dimension. Plots with *R. alpinus* were selected to cover a gradient from very low (minimum 5%) to very high cover of the invader and to represent spatial distribution of the species within the study area. Control plots were located spatially very close to the paired plots with *R. alpinus*; most often they were adjacent or within distance of 2 m, with highly similar habitat conditions. The cover of the invader in the control plot was allowed to be at a maximum of 1%. Examined plots were distributed at elevations ranging from 991 m to 1497 m a.s.l. (interquartile range 1110–1295 m a.s.l.).

**Table 1:** List of localities in the Tatra National Park from which *R. alpinus* plots were sampled

No.	Locality	Number of sampled *R. alpinus* plots	Elevational range of sampled plots (m a.s.l.)	Latitude (N)	Longitude (E)
1	Polana Przysłop	7	1177–1227	49.25747°	19.80455°
2	Polana Jaworzyna	5	1293–1303	49.25430°	19.79884°
3	Polana Jaworzyna Lejowa	3	1008–1018	49.26408°	19.84343°
4	Polana Huty Lejowe	5	991–1002	49.26317°	19.84553°
5	Polana Kuca	4	1167–1198	49.26146°	19.83581°
6	Kominiarski Przysłop	6	1129–1139	49.25649°	19.85880°
7	Niżnia Kominiarska Polana	13	1116–1147	49.25388°	19.84973°
8	Polana Upłaz	10	1288–1336	49.25117°	19.88256°
9	Stara Polana	1	1113	49.23388°	19.85728°
10	Polana Smytnia	7	1089–1102	49.23195°	19.86007°
11	Mała Polanka Ornaczańska	2	1104–1105	49.22741°	19.85830°
12	Wielka Polana Ornaczańska	7	1112–1145	49.22305°	19.85520°
13	Niżnia Smreczyńska Polana	2	1186–1187	49.22522°	19.86814°
14	Wyżnia Smreczyńska Polana	5	1247–1268	49.22127°	19.86697°
15	Niżnia Tomanowa Polana	2	1341–1343	49.22419°	19.88665°
16	Wyżnia Tomanowa Polana	2	1387–1388	49.22317°	19.89041°
17	Polana Olczyska	8	1087–1093	49.26697°	19.99981°
18	Hala Gąsienicowa	2	1492–1497	49.24340°	20.00850°
19	Włosienica	2	1317–1319	49.21342°	20.08050°
20	Morskie Oko	7	1395–1413	49.20166°	20.07024°

Species composition was surveyed for each plot, and phytosociological relevés were made using Braun-Blanquet’s method and a six-point plant cover scale (Kent 2012), whereas the abundance of *R. alpinus* was estimated in terms of percentage cover (Supplementary Table 1). The nomenclature of vascular plants followed Mirek et al. (2002). In the space-for-time substitution approach, 55 plots with *R. alpinus* cover of at least 50% (dominance of the species) were selected (hereafter, referred to as invaded plots) together with their paired control plots (hereafter, referred to as uninvaded plots); 36 sites were located in the lower montane belt and 19 in the upper montane belt. Analyses of diversity and vegetation composition in plots differing in the extent of *R. alpinus* cover were performed for all 100 plots with *R. alpinus*.

### Data analysis

For each plot, we calculated the plant species richness (*S*) as the number of vascular plant species per plot, Shannon diversity index (*H´*) as *H´* = − ∑*p*_*i*_ × ln *p*_*i*_, Simpson diversity index (*D*) as *D* = 1 – ∑*p*_*i*_^2^ and Pielou’s evenness index (*J*) as *J* = *H´* / ln *S*, where *p*_*i*_ is the proportion of species *i* per plot (Hill 1973). For diversity calculations, Braun-Blanquet cover-abundance values +, 1, 2, 3, 4 and 5 were transformed to 0.1, 2.5, 15.0, 37.5, 62.5 and 87.5, respectively (Wildi 2010). *R. alpinus* was not included in the data set used in the calculation of the diversity indices in order to evaluate the impact of the invader on the remaining species. To assess the impact of the invasion by *R. alpinus* on resident vegetation, we compared invaded and uninvaded plots with respect to species richness, diversity and evenness measures. This comparison was made also separately for sites located in the lower and the upper montane belts. As statistical distributions of most of these diversity indices deviated from the normal distribution significance of differences in diversity measures between invaded and uninvaded plots, they were tested by the Wilcoxon signed-rank two-sided test. To examine the impact of an increasing invader cover on the diversity of plant communities, simple linear regression and quadratic regression were applied. In this approach, diversity indices were modelled, as a response variable, by percentage cover of *R. alpinus* in a plot. The effect of *R. alpinus* cover on species diversity was tested using simple linear regression: *Y = a + b*_*1*_ *× *(*R. alpinus cover*) and regression with a quadratic term added: *Y = a + b*_*1*_ *× *(*R. alpinus cover*)* + b*_*2*_ *× *(*R. alpinus cover*)^*2*^, where *a* denotes an intercept, and *b*_*1*_ and *b*_*2*_ denote regression coefficients. Linear and quadratic models were fitted to *R. alpinus* cover gradient, and the best model was selected based on an analysis of variance (ANOVA) test, where a significant *F* statistic expressed significant improvement of the linear model once the quadratic term was added (Dalgaard 2008). In analogous way, we examined whether the impact of *R. alpinus* on diversity of invaded vegetation differed across elevations; in this analysis diversity indices were modelled by elevation in simple linear and quadratic regressions, both run separately for invaded and uninvaded plots.

The impact of invasion by *R. alpinus* on species composition was examined with indirect gradient analysis, Detrended Correspondence Analysis (DCA), performed for 55 pairs of invaded-uninvaded plots. In DCA analysis, rare species occurring only in one or two plots (relevés) were excluded from subsequent analyses (Legendre and Legendre 2012). Also, *R. alpinus* was excluded from this analysis because the aim of the analysis was to examine changes in the composition of the remaining species of resident vegetation. Species abundances were expressed in the original Braun-Blanquet scale (+, 1, 2, 3, 4, 5), with transformation of ‘+’ into a value of 0.1. This approach was applied to down-weight the influence of accidental high abundances of species on the ordination result. To understand the ecological shift in the species composition of invaded plots in relation to uninvaded plots, we superimposed on the ordination diagram environmental factors, expressed by elevation of investigated sites and mean Ellenberg’s indicator values (EIVs), fitted post hoc to the DCA ordination. EIVs for light, soil nutrients, soil moisture and soil acidity (Ellenberg et al. 1992) were assigned to all species, if available, and their mean values for each plot were calculated. Only these factors, which proved to be significant in the permutation test (*n* = 999) at the 0.05 significance level, were presented on the ordination diagram. The effect of the invasion on the species composition was assessed by comparison of the DCA site scores of the pairs of invaded-uninvaded plots along the first and the second ordination axes, applying the Wilcoxon signed-rank test.

All statistical analyses were performed in R version 3.4.2 (R Core Team 2017). To compute diversity indices and perform DCA analysis, the Community Ecology Package ‘vegan’ was used (Oksanen et al. 2017).

## Results

### Species richness, diversity and evenness

**Fig. 2:**
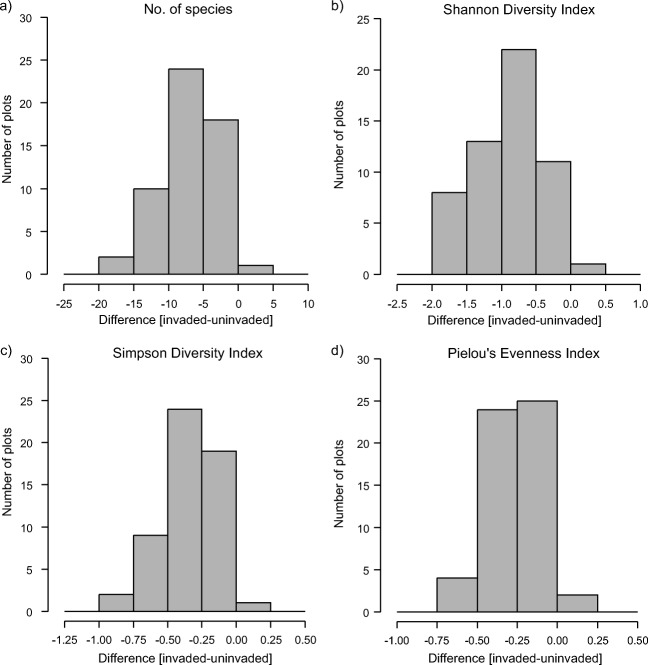
Distribution of paired differences in diversity indices between invaded and uninvaded plots

Invaded plots dominated by *R. alpinus* had significantly reduced species richness and diversity compared to uninvaded control plots (Fig. 2). Uninvaded plots harboured an average of 19 species, while the invaded plots harboured 13 (Table 2). This difference was highly significant (*V* = 1.5, *p* < 0.001, *n* = 55). The weighted mean decline in the number of species in pairs of invaded-uninvaded plots was 37.7%. Uninvaded plots most often had 5 to 10 species fewer compared to their uninvaded control plots (Fig. 2a). In total, 89 and 113 species were recorded in invaded and uninvaded plots respectively (Table 2).

**Table 2:** Number of species, Shannon diversity index *H*´, Simpson diversity index *D* and Pielou’s evenness *J*, as recorded in the studied plots

	*Rumex* plots	Control plots	Wilcoxon test	*P* value
Total number of species	89	113		
Plot species richness				
*Median*	13	19	V = 1.5	p < 0.001
*Min*	5	11		
*Max*	20	31		
Shannon Diversity Index	0.89	1.74	V = 2	p < 0.001
Simpson Diversity Index	0.45	0.77	V = 15	p < 0.001
Pielou’s Evenness Index	0.37	0.62	V = 6	p < 0.001

Vegetation dominated by *R. alpinus* exhibited significantly lower values of Shannon diversity. For the invaded plots, this index value constituted 51.1% of the diversity of the uninvaded plots, yielding a highly significant difference (*V* = 2, *p* < 0.001, *n* = 55) (Fig. 2b; Table 2). Simpson diversity was also significantly reduced in the invaded plots at a value of 58.4% of the uninvaded plots’ diversity (*V* = 15, *p* < 0.001, *n* = 55) (Fig. 2c; Table 2). Similarly, Pielou’s evenness index for the invaded plots constituted 59.7% of the index calculated for the uninvaded plots; this difference proved to be significant as well (*V* = 6, *p* < 0.001, *n* = 55) (Fig. 2d; Table 2).

**Fig. 3:**
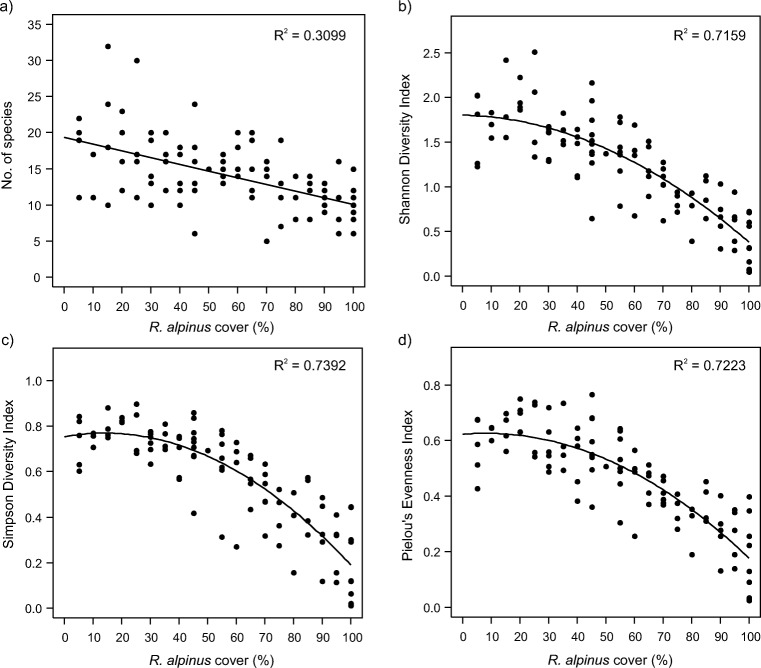
Variation in plot diversity indices based on increasing *R. alpinus* coverage in the studied plots; **a** number of species, **b** Shannon diversity index *H*´, **c** Simpson diversity index *D*, **d** Pielou’s evenness index *J*

Along with an increase in the abundance of *R. alpinus* in vegetation, significant changes in species richness and diversity were observed. Species richness was better fitted to *R. alpinus* coverage by a linear model than a model with a quadratic term added for the invader percentage cover (Fig. 3a; Table 3). The number of species calculated by applying a simple linear regression decreased from 18.6 at 5% *R. alpinus* coverage to 10.0 at 100% coverage, resulting in a 46% reduction in species richness. The three other investigated measures of species diversity were significantly better fitted by models with a quadratic term for *R. alpinus* coverage; however, their maxima were observed at the lowest values of *R. alpinus* coverage (Fig. 3b–d; Table 3). Shannon diversity index (Fig. 3b) decreased in value from 1.796 at 5% *R. alpinus* coverage to 0.380 at 100% coverage. The Simpson diversity index (Fig. 3c), after a very slight increase from 0.763 at 5% *R. alpinus* coverage to 0.770 at 14% coverage, decreased to 0.190 at 100% coverage. Pielou’s evenness index (Fig. 3d) decreased from 0.626 at 5% *R. alpinus* coverage to 0.177 at 100% coverage.

**Table 3:** Regression summaries for species diversity indices fitted to *R. alpinus* coverage in the studied plots

*Y*	*a (intercept)*	*b*_*1*_	*b*_*2*_	*F*_*model*_	*P value*
*Species number*	19.325 × 10^+00^	−9.314 × 10^−02^	–	0.90	0.3457
*Shannon Diversity Index*	1.803 × 10^+00^	−7.460 × 10^−04^	−1.348 × 10^−04^	12.65	0.0005
*Simpson Diversity Index*	7.541 × 10^−01^	2.216 × 10^−03^	−7.860 × 10^−05^	26.92	< 0.0001
*Pielou’s Evenness Index*	6.225 × 10^−01^	8.905 × 10^−04^	−5.345 × 10^−05^	19.44	< 0.0001

Significant *F*_*model*_ statistic (*P value* < 0.05) expressed significant improvement of the linear model once the quadratic term was added

**Fig. 4:**
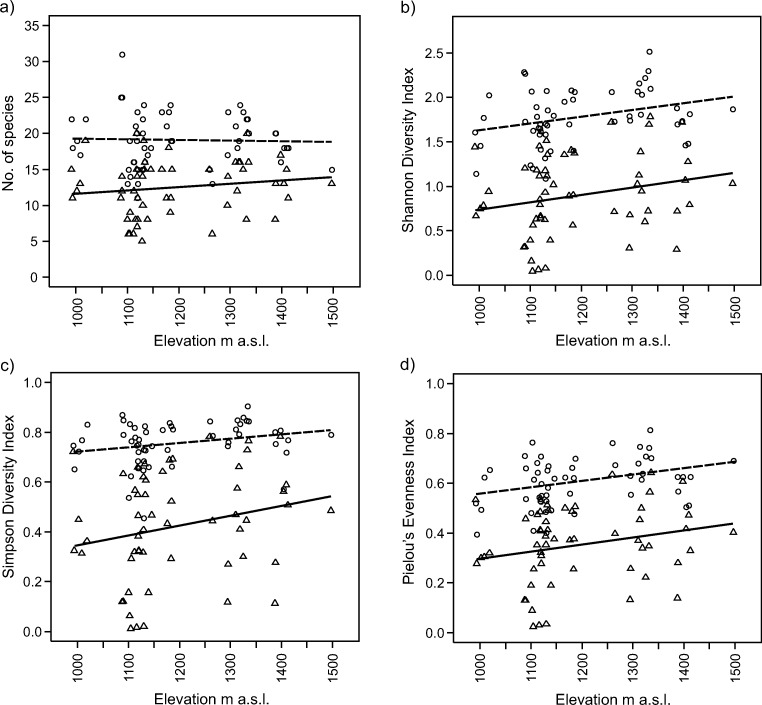
Elevational variation in plot diversity indices; triangles – *R. alpinus*-invaded plots, circles – uninvaded plots; solid line – regression line for invaded plots, interrupted line – regression line for uninvaded plots

Separate invaded and noninvaded plot regression analyses of elevation changes in species diversity revealed the existence of only slight straight-line elevation trends (Fig. 4). Quadratic regression models were not better fitted for diversity indices than linear models, though the slopes of linear regressions were not statistically significant. With an increase in elevation, there was a slight trend of increase in all diversity measures, both for invaded and uninvaded plots, with the exception of mean number of species per plot (Fig. 4a), which was almost constant across all elevations. The slopes of regression lines for invaded and uninvaded plots were nearly parallel for Shannon diversity and Pielou’s evenness indices (Fig. 4b, d), suggesting similar impacts of *R. alpinus* across all elevations. However, analysis of the Simpson diversity index signified an interaction between responses in diversity across all elevations. Still, low elevation differences between invaded and uninvaded plots in the Simpson index were larger than at high elevations (Fig. 4c).

### Species composition

**Fig. 5:**
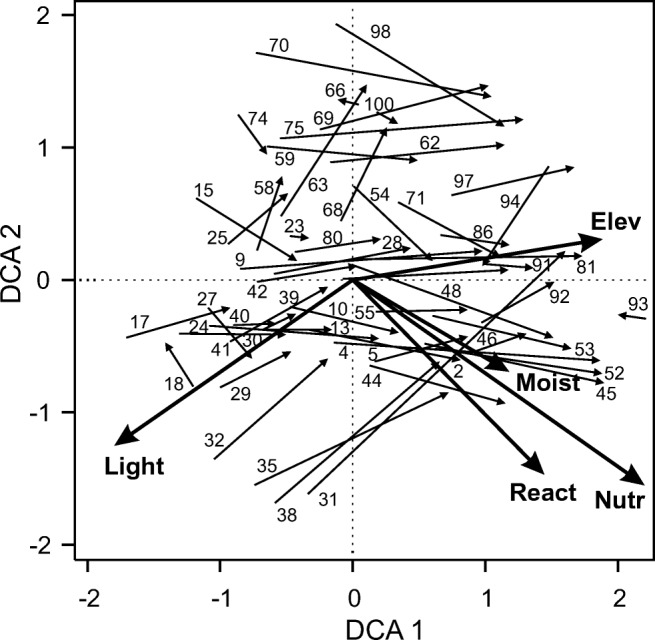
DCA ordination diagram of invaded and uninvaded plots; arrows start at ordination scores of uninvaded plots and point towards paired invaded plots, indicating a shift in species composition. Lengths of axes are 3.926 and 3.617 for axis 1 and axis 2 respectively. Numbers on the plot correspond to plot numbers in Supplementary Table 1

**Fig. 6:**
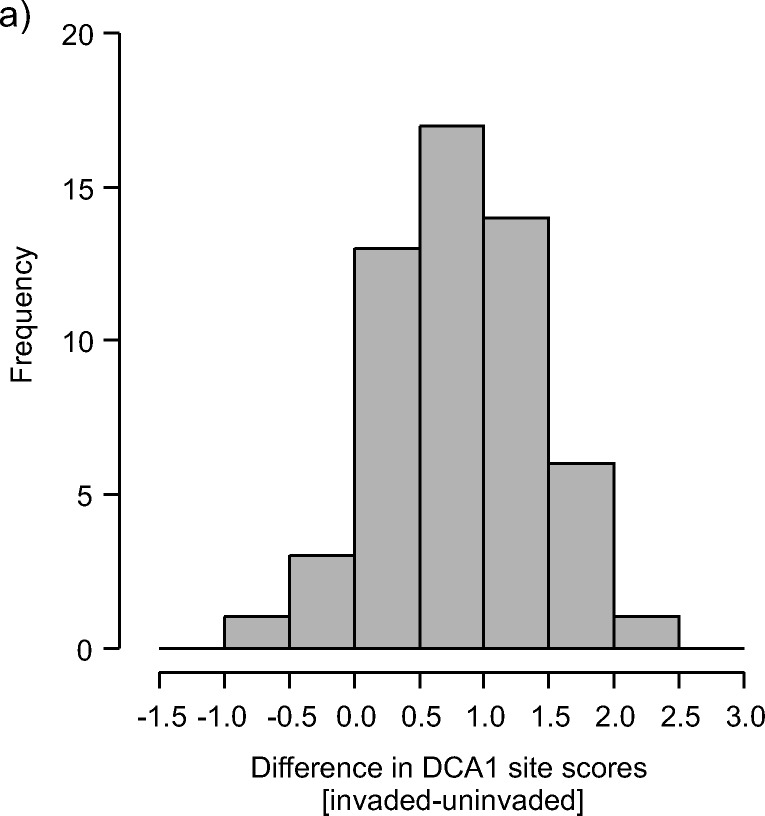
Differences in DCA ordination scores for pairs of invaded-uninvaded plots along the first ordination axis

Increased *R. alpinus* abundance caused changes in the floristic composition of invaded vegetation. Detrended Correspondence Analysis revealed that the majority of invaded vegetation plots, as compared to uninvaded plots, shifted along the first DCA axis to the right, towards higher values of nutrient, moist and reaction EIVs and lower values of light EIV (Figs. 5; 6). This shift of DCA scores along the first axis was considerable (median = 0.786, *V* = 1507, *p* < 0.0001, *n* = 55). Along the second DCA axis, no significant shift in scores of invaded samples, as compared to uninvaded, was observed (median = 0.038, *V* = 881, *p* = 0.357, *n* = 55). Plot ordination along the first DCA axis was also significantly correlated with elevation.

The most frequent species in the *R. alpinus*-dominated vegetation plots, present in at least 50% of plots, included *Rumex alpestris* (84% frequency), *Deschampsia caespitosa* (67%), *Chaerophyllum hirsutum* (64%), *Veronica chamaedrys* (62%), *Stellaria nemorum* (60%), *Urtica dioica* (56%) and *Ranunculus repens* (53%) (Supplementary Table 1). The species most negatively affected by invasion, which avoided vegetation dominated by *R. alpinus* despite its presence in uninvaded control plots, included *Achillea millefolium* and *Stellaria graminea* (each at a 36% avoidance rate in pairs of invaded-uninvaded plots), *Festuca pratensis* (34% avoidance rate), *Festuca rubra* and *Agrostis capillaris* (each 33%), *Ranunculus acris* and *Senecio subalpinus* (each 31%), *Alopecurus pratensis* (29%), *Hypericum maculatum* (27%), *Alchemilla* sp. (25%), *Trifolium repens* and *Poa trivialis* (each 24%), *Ranunculus repens* (22%) and *Poa pratensis*, *Carex ovalis* and *Galeopsis tetrahit* (each 20%). More associated with the invaded rather than uninvaded vegetation were *Urtica dioica* and *Stellaria nemorum*, species present in 16% and 15% of the invaded vegetation samples respectively, despite simultaneous absences in their paired uninvaded controls.

## Discussion

This study presents results of the potential impacts of the troublesome weed *R. alpinus* on plant diversity in invaded vegetation. Invasion of mountain pastures by *R. alpinus* is associated with a considerable reduction in plant diversity. All the investigated diversity measures (species richness, Shannon and Simpson diversity indices and Pielou’s evenness index) had significantly lower values in *R. alpinus*-dominated plots compared to uninvaded plots. The mean number of species per plot decreased steadily with an increase in *R. alpinus* coverage. Other diversity measures, such as Shannon and Simpson diversity indices and Pielou’s evenness values, which were better suited for curvilinear regression, did not considerably change up to ~30% of *R. alpinus* coverage, but a further increase in *R. alpinus* coverage led to a steep decline in diversity measures. DCA analysis of species composition revealed significant shifts in pairs of invaded-uninvaded plots. These shifts can be interpreted as change in the invaded vegetation composition, which resulted from the shared increase of more shadow-tolerant and higher soil base-, nutrient- and moist-demanding species, as indicated by Ellenberg’s indicator values in the DCA ordination.

It is worth noting that the decline in species richness of the invaded vegetation, as compared to the uninvaded, by as much as the 37.7% found in our study agrees with the results of Hejda et al. (2009), who found a 39.1% decrease in the number of species in *R. alpinus* invaded vegetation in the Czech Republic. According to these authors, out of the twelve other investigated invasive species only four had greater impacts than *R. alpinus* on species richness, namely the three *Fallopia* species *F. sachalinensis*, *F. japonica*, and *F.* × *bohemica*, and *Heracleum mantegazzianum*. This suggest that *R. alpinus* is one of the species which most negatively influences vegetation in central Europe. As explained by Klimeš (1992), competitive dominance of *R. alpinus* can be attributed to its ability to form dense canopies, which are penetrated only by 3–5% sunlight. Under such canopy, the vitality and reproduction of companion species are fairly limited. Moreover, *R. alpinus* is able to suppress neighbouring meadow vegetation with its large leaves; on stand margin, the petioles, which can grow up to 90 cm in length, are bent towards the outside of the stand and cast shadows up to 70 cm from the shoot base (Klimeš 1992).

As a plant with populations known to persist for several decades (Ellenberg 2009; Klimešová et al. 2013), alpine dock is a very difficult species to control by non-chemical methods and is therefore a problematic weed, especially in protected areas such as nature reserves and national parks. For example, Šilc and Gregori (2016) found that only manually excavating plants or covering them with black polyethylene foil for at least two seasons almost completely removed the biomass and cover of *R. alpinus*. Mowing reduced the cover of alpine dock to 50%, but was effective only when applied frequently and regularly. Controlling alpine dock with grazing animals was found to be ineffective as well, because animals could only graze on *R. alpinus* in grasslands where the species was scattered, while large monodominant stands and patches of alpine dock were avoided by grazing animals (Šilc and Gregori 2016).

In this study, we analysed the relationships between diversity indices in invaded and uninvaded plots, as well as in plots with differing *R. alpinus* coverage, applying approaches that are often used to investigate potential impacts of invasive plants on vegetation (e.g. Hejda and Pyšek 2006; Hejda et al. 2009; Kiełtyk 2014; Künzi et al. 2015; Diekmann et al. 2016). However, it should be acknowledged that from a formal point of view, this study cannot render a definitive statement as to the causes of the observed diversity pattern, nor to what degree the observed diversity pattern was driven by *R. alpinus* or some other, unmeasured factors. Further experimental studies on permanent plots conducted for several years will contribute to enhanced assessment of the overall impact of *R. alpinus* on resident vegetation.

## Electronic supplementary material

Supplementary Table 1 Vegetation table of the herb layer of 100 pairs of plots invaded (i) and uninvaded (u) by *R. alpinus (XLSX 109 kb)*
